# Study of Flow Characteristics of Gas Mixtures in a Rectangular Knudsen Pump

**DOI:** 10.3390/mi10020079

**Published:** 2019-01-24

**Authors:** Zhijun Zhang, Xiaowei Wang, Lili Zhao, Shiwei Zhang, Fan Zhao

**Affiliations:** 1 School of Mechanical Engineering and Automation, Northeastern University, Shenyang 110819, China; xiaowwang812@163.com (X.W.); shwzhang@mail.neu.edu.cn (S.Z.); nvacuum_zhaofan@163.com (F.Z.); 2 School of Mechanical Engineering, Shenyang University, Shenyang 110044, China; zhaolili0214@163.com

**Keywords:** Knudsen pump, thermally induced flow, gas mixtures, direct simulation Monte Carlo (DSMC), microfluidic

## Abstract

A Knudsen pump operates under the thermal transpiration effect or the thermal edge effect on the micro-scale. Due to the uneven temperature distribution of the walls in the channel axis direction or the constant temperature of the tips on the walls, directional thermally-induced flow is generated. In this paper the Direct Simulation Monte Carlo (DSMC) method is applied for N_2_–O_2_ gas mixtures in the ratios of 4:1, 1:1, and 1:4 with different Knudsen numbers in a classic rectangular Knudsen pump to study the flow characteristics of the gas mixtures in the pump. The results show that the changing in the gas physical properties does not affect the distribution of the velocity field, temperature fields, or other fields in the Knudsen pump. The thermal creep effect is related to the molecular mass of the gas. Even in N_2_ and O_2_ gas mixtures with similar molecular masses, N_2_ can be also found to have a stronger thermal creep effect. Moreover, the lighter molecular weight gas (N_2_) can effectively promote the motion of the heavier gas (O_2_).

## 1. Introduction

It is well known that the thermally-induced flow of rarefied gas is generated by the temperature gradient along the walls of the Knudsen pump and that the gas is driven to flow from the low-temperature side to the high-temperature side. That is the basic mechanism of the Knudsen pump which was first put forward by Danish physicist Martin Knudsen in 1909 [1]. The Knudsen pump can provide consistent gas flow and has the advantage of having of no moving parts, a simple structure, ease of operation, long life span, low energy consumption, and wide energy sources. It is widely applied in Micro Electro Mechanical Systems (MEMS) such as gas separators [2,3], gas analysis [4,5,6], micro combustors [7,8], and micro-air vehicle systems [9,10].

**Figure 1:**
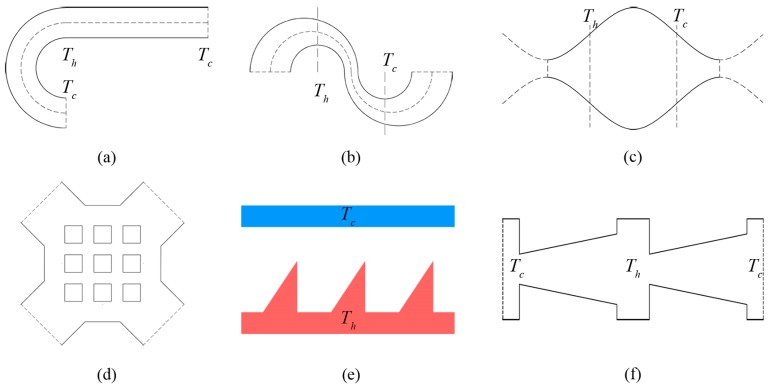
Common Knudsen pump channel structures: (**a**) Curve-straight channel, (**b**) double-curves channel, (**c**) sinusoidal channel, (**d**) matrix channel, (**e**) ratchet channel, (**f**) taper channel.

The classic rectangular Knudsen pump is composed of a series of alternately connected wide and narrow micro-channels [1]. A tangential temperature gradient appears by imposing high-temperature and low-temperature heat resources for the two ends of the wide channels respectively. This generates a thermal creep effect for the gas flow. In recent years, with the development of materials technology and micro-machining technology, the pump structure can now be produced by using poly-silicon material, and using the inter-molecular gaps in porous materials such as aerogel membranes [10,11,12], mixed cellulose ester (MCE) [13,14], zeolite [15,16], porous ceramics [17,18] and Bi_2_Te_3_ [19,20] to construct the flowing channel of the Knudsen pump. Since the rectangular Knudsen pump has been proposed, many structures for the channel were successively designed and studied (Figure 1), including the sinusoidal micro-channel [21], matrix micro-channel [21], curved micro-channel with different curvature radii [22], alternately connected curved and straight micro-channel [23,24], tapered micro-channel [25], and ratchet micro-channel [26,27,28,29].

It is well known that the Boltzmann equation is the basic equation for solving the continuous, transition, and free-molecular regimes. It has been developed in mathematical methods such as the moment method and model equation in recent years. Chapman-Enskog solution is the most important representative of the moment method, whose first-order solution is the Navier–Stokes–Fourier (NSF) equation [30,31]. By adding velocity slip and temperature jump conditions, the Chapman-Enskog solution can be applied to the rarefied gas flow within a small Knudsen number. However, the regularized 13-moment equations [32] can be used for the rarefied gas flows with a large Knudsen number. The model equation simplifies the collision integral in the Boltzmann equation. The most famous model equations are the BGK model introduced by Bhatnagar, Gross and Krook [33] and McCormack model [34]. The BGK model is well applied to the study of transport characteristics of gas mixtures in micro-channels [35,36]. The McCormack model also shows good consistency with experimental results in studying the flow state of gas mixtures [37]. The Direct Simulation Monte Carlo (DSMC) method is a direct numerical solution of the Boltzmann equation, which eliminates the disadvantages in the mathematical solution of the Boltzmann equation. Although the DSMC method requires a large amount of internal storage space and long calculation time, its calculation results are highly consistent with experimental results [38,39,40].

Compared with other study methods [21,22,23,24,41,42,43,44,45,46], the DSMC method is widely used for heat and mass transfer in micro-channels [47,48,49,50]. There are many studies that apply the DSMC method in the flow of gas mixtures [51,52,53]. It is found that Knudsen pump shows good capability in gas separation by the simulation of DSMC [54]. While in the studies of gas flow in Knudsen pumps, the method of DSMC is widely employed [28,29,55]. For example, DSMC is used to study and simulate flow patterns of the gas in rectangular channels [55] and ratchet channels [28,29]. The studies of Knudsen pumps have generally been focused on innovation of the structure, optimization of performance, and practical application. The gas used in simulations is mostly monatomic noble gas. However, gas mixtures have been more widely applied than single gases, and the proportions of the noble gas in the air are very small. Moreover, the size of the micro-channel has already reached the nanometer level, ensuring that the Knudsen pump operates normally under atmospheric pressure.

In present study, the flow characteristics of gas mixture of N_2_ and O_2_ in Knudsen pump are simulated with the DSMC method. A classic rectangular channel is applied, that is more common and convenient to machine. The problem statement and the numerical method are presented in Section 2. The simulation results for the gas mixtures of N_2_ and O_2_ in three different ratios are discussed in Section 3, and the conclusions are in Section 4.

## 2. Problem Statement and Numerical Method

### 2.1. Problem Statement

**Figure 2:**
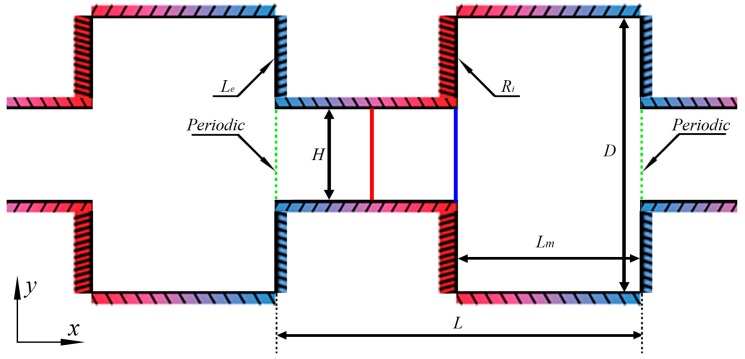
Configuration and geometric parameters.

The configuration consists of the alternately connected narrow and wide micro-channels shown in Figure 2. A periodic structure is well-established in the *x*-direction. For decreasing the calculation amount and improving the simulation efficiency, a basic unit presented by the green dash-dotted line in Figure 2 is extracted. The periodic boundary conditions on the inlet and outlet are used for the recurrence of the physical structure. When the simulation particles pass through these two boundaries, except for the change of the position in *x*-direction ($x_{\mathrm{Inlet}}=x_{\mathrm{Outlet}}-L$), all the other parameters such as distribution function, velocity, temperature, acting force Φ are definitely equal, that is $\Phi_{\mathrm{Inlet}}=\Phi_{\mathrm{Outlet}}$.

The distance between the two walls of the narrow channel is indicated by *H*, and the distance (*D*) between the two walls of the wide channel is triple that of the narrow channel. The length of the basic unit is *L*; the length of the narrow channel and the wide channel in the *x*-direction are both equally *L_m_*, which is half of *L*.

The configuration, temperature, are geometric parameters of the rectangular channel are *H* = 1 μm, *D* = 3*H* = 3 μm, *L* = 4 μm, and *L_m_* = *L*/2 = 2 μm, respectively. In consideration of the thermal resistance of the materials in practice and the high heat flux density in the micro-scale, the reference temperatures of the cold walls and hot walls are, respectively, $T_{c}=225\;K$ and $T_{h}=375\;K$ [27,28]. In the paper, all parameters are only for the two-dimensional surface. Assume that the third dimension is infinite (defining the property as “empty”), and there is no influence of the scale effect in the z-direction on the flow characteristics. However, the OpenFOAM software (version 4.1, OpenCFD Ltd, Bracknell, England, UK) is limited to three-dimensional models, and to enable comparison to the mass flow rate in [28], the width in the z-direction is also considered as 20 μm in modeling.

The Knudsen number [40,56]:

(1) $$Kn=\frac{\lambda }{H}=\frac{2(5-2\omega )(5-2\omega )}{15H}{\left(\frac{M}{2\pi kT_{m}}\right)}^{1/2}\left(\frac{\mu }{\rho }\right)$$

is defined by the characteristic dimension *H*, the distance between the two walls of the narrow channel. In Equation 1, *P* is the pressure. The mean temperature is $T_{m}=\left(T_{h}+T_{c}\right)/2$ and $M=CM_{a}+(1-C)M_{b}$ is the average molar mass of the mixture. The number densities of the components are denoted by $n_{a}$ and $n_{b}$. *C* is the molar fraction of the first gas component. The dynamic viscosity μ is calculated from the Chapman-Enskog theory. It can be written as $\mu =\mu_{a}+\mu_{b}$, where $\mu_{a}$ and $\mu_{b}$ are assumed to be dependent on the temperature $T_{m}$ according to the law [35,51].

### 2.2. DSMC Method

The DSMC method is based on three basic dynamic theories of rarefied gas, the ergodic assumption, binary collision assumption, and the molecular chaos assumption. The flowing characteristics of the real gas molecules in the micro-channels are represented by a set of simulation particles in the process of simulation. With its movements, inter-molecular collisions and the interaction of the boundary walls, some information of the simulation particles can be stored in the computer, including position, velocity, internal energy, and so on. The simulating process for the gas flow is achieved by applying statistics to obtain the average information of the simulation particles in all cells to represent the macroscopic variation.

Additionally, the time step of DSMC method should be far lower than the mean collision time. Therefore, the real processes of the molecular free movement and the inter-molecular collisions are decoupled into two consecutive steps. It is assumed that molecules are in uniform rectilinear motion in the original direction within one time step size. If collisions with the walls occur in the process of the free movement, the collision will be calculated first, and the post-velocity will be used for the free movement and inter-molecular collisions. Finally, the microscopic variables of all simulation particles in the cells can be used to depict the macroscopic physical quantities using statistics. There are many molecular models depicting inter-molecular collisions. The most famous are the hard sphere (HS), variable hard sphere (VHS), variable sphere (VS) and variable soft sphere (VSS) models [57]. The variable hard sphere (VHS) model is widely used in studies of the Knudsen pump. The VHS model is adopted in this work. The post-collision velocities of a colliding pair of molecules can be found in [38,39,40]. In addition, the no time counter (NTC) scheme [40] is used to ensure the correct number of collisions, which is consistent with the analytical theory.

For the boundary conditions, the constant cold temperature $T_{c}$ and the constant hot temperature $T_{h}$ are respectively exerted on the left and right walls of the wide channel (*L_e_* and *R_i_* in Figure 2). The wall can be made of high thermal conductivity material. Therefore, because of the heat transfer property, the positive constant temperature gradient is applied for the walls of the narrow channels. Additionally, the negative constant temperature gradient is applied for the walls of the wide channels in the actual applications. The walls are adopted as to completely diffuse reflection. All the particles colliding with the walls are diffusely reflected according to Maxwellian velocity distribution.

### 2.3. Code Validation

**Figure 3:**
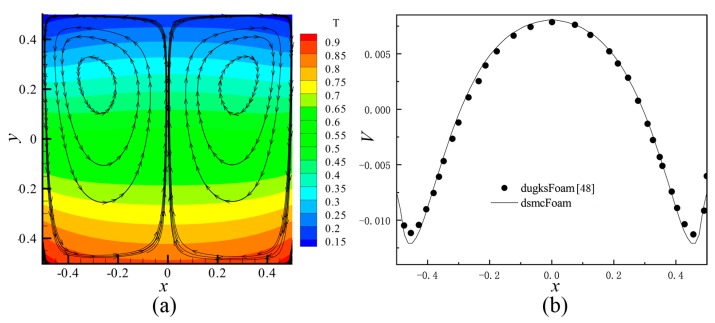
The simulation results of square cavity with dsmcFoam. (**a**) Temperature contours and velocity streamlines at *Kn* = 0.1 by dsmcFoam. (**b**) Profile of the *V* component of the velocity on horizontal line, passing through the center of the left primary vortex at *Kn* = 0.1. In (**b**), black points show the results extracted from [43], and the curve shows the results of dsmcFoam.

In the present study, an open source Direct Simulation Monte Carlo (DSMC) code, dsmcFoam [56,58] was employed. This solver has been tested in a lot of cases, such as 2D flow over a flat plate and a cylinder, and 3D supersonic flows over complex geometries. The dsmcFoam shows very good agreement with data provided by both analytical solutions and other contemporary DSMC codes. Furthermore, Shahabi et al. [29] applied this solver to study the physical mechanism of the thermally induced flow in ratchet Knudsen pump. In order to verify the feasibility of dsmcFoam in dealing with the thermally induced flow problems more clearly, thermally induced flow in square cavity was simulated in this paper. The same parameters were used, and the results were compared with the discrete unified gas kinetic method, dugksFoam [43]. It can be seen that both results agree well with each other, as shown in Figure 3 (the results of dugksFoam is not shown in Figure 3a).

### 2.4. Grid, Particle and Time Step

Grid (or cell) size and the number of simulation particles in the cell are two main factors influencing the calculating efficiency and accuracy. Bird [38,39,40] pointed out that the cell size Δx should not exceed 1/3 of the mean free path λ, and the number of simulation particles $N^{\prime }$ in every cell should range from 20 to 30 which assumes that the number is averagely distributed.

(2) $$N^{\prime }=\frac{F_{N}}{N_{C}}$$

where $N_{C}$ is the number in the cell and $F_{N}$ is the sample number, which relies on the ratio of the number of real molecules *N* to equivalent particle number *E*_p_:

(3) $$F_{N}=\frac{N}{E_{p}}=\frac{P/kT_{m}}{E_{p}}$$

**Figure 4:**
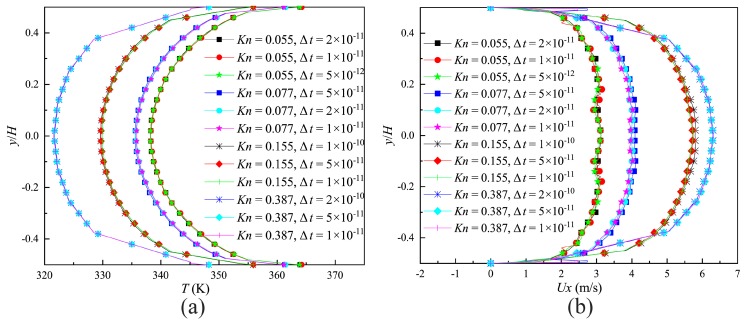
Calculation results of different time steps. (**a**) Velocity distributions on the outlet of the narrow channel; (**b**) temperature distributions on the outlet of the narrow channel.

In terms of the gas mixtures, the cell size Δx should be smaller than 1/3 of the minimum mean free path ($\lambda_{\min}=\min(\lambda_{a},\lambda_{b},\ldots )$). The relation between the time step Δt and the mean collision time (MCT) or the mean transit time (MTT) can be represented as follows [59]:

(4) $$0.005\leq \Delta t/t_{0}\leq 0.5$$

where, $t_{0}=\lambda_{\min}/v_{m}$ is the MCT, $v_{m}=\sqrt{2kT_{m}/m}$ is the most molecular probable speed. The effect of different time steps on the velocity and temperature distributions on the surface of the narrow channel outlet (indicated by the blue full line in Figure 2) compared in Figure 4a,b. It is demonstrated that a time step of $\Delta t<\lambda_{\min}/(3v_{m})$ could provide the time-step independent solutions.

Besides, in the actual simulation, when the number of simulation particles in the cell is over 15 and the cell size is $\lambda_{\min}/3$, the results of the calculation do not have significant differences, as shown in Table 1. After comprehensive consideration, in this study, the number of simulation particles in the cell is around 15 and the cell size is about $\lambda_{\min}/3$. With the rarefied degree of the rarefied gas increasing, 1/3 of the mean free path will probably exceed the size of the geometric model. Therefore, to avoid this circumstance, the oversize cell size hampers the normal division of the mesh. When the Knudsen number is over 0.387, the cell size remains (equal to the size for *Kn* = 0.387).

## 3. Results and Discussion

This section mainly discusses the flowing characteristics of the gas mixtures of N_2_ and O_2_ in the ratios of 4:1, 1:1 and 1:4 in the rectangular channel Knudsen pump. The flow field, distribution of temperature gradient, distribution of velocity, and mass flow rate are thoroughly studied. The physical properties of the gases for the simulation particles of N_2_ and O_2_ in the VHS model are listed in Table 2.

### 3.1. Velocity and Temperature Distribution

**Figure 5:**
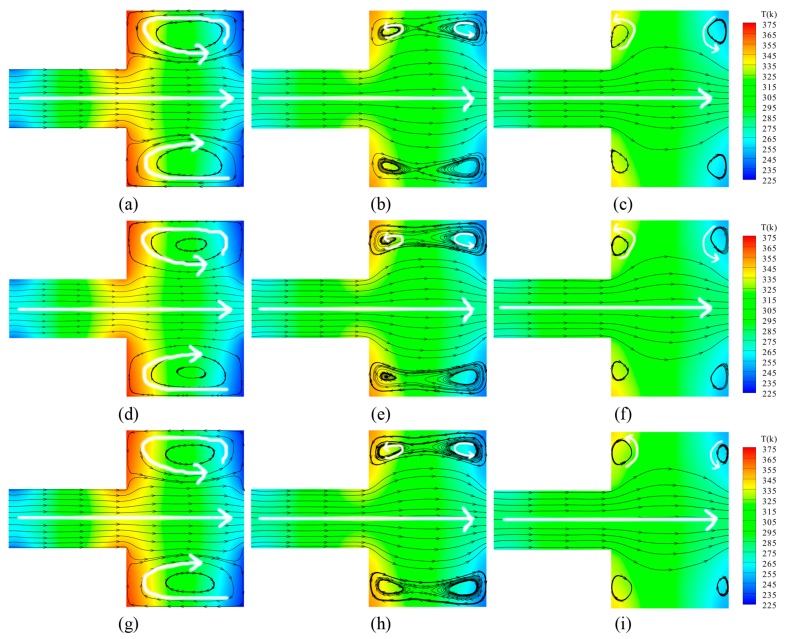
Velocity streamlines and temperature contours for different ratios of N_2_ and O_2_ and different Knudsen numbers. (**a**) N_2_ and O_2_ = 4:1, *Kn* = 0.055; (**b**) N_2_ and O_2_ = 4:1, *Kn* = 0.387; (**c**) N_2_ and O_2_ = 4:1, *Kn* = 3.87; (**d**) N_2_ and O_2_ = 1:1, *Kn* = 0.055; (**e**) N_2_ and O_2_ = 1:1, *Kn* = 0.387; (**f**) N_2_ and O_2_ = 1:1, *Kn* = 3.87; (**g**) N_2_ and O_2_ = 1:4, *Kn* = 0.055; (**h**) N_2_ and O_2_ = 1:4, *Kn* = 0.387; (**i**) N_2_ and O_2_ = 1:4, *Kn* = 3.87.

For N_2_ and O_2_ in the three different mixed ratios, the representative velocity and temperature distribution in the rectangular channel for *Kn* = 0.055, *Kn* = 0.387, and *Kn* = 3.87 are illustrated respectively in Figure 5.

By observing the figures, it can be seen that gases flow forward from left to right in the narrow channels without differences, though the Knudsen number increases. However, large differences are triggered in the wide channels, and the rarefied degree of the gas is enhanced. A larger circular-flow vortex appears respectively in the upper side and the lower side of the wide channel for *Kn* = 0.055. When the Knudsen number increases, the velocity stream of gases near the central axis expands towards the upper side and the lower side. Within the larger vortex, two anticlockwise secondary vortexes are generated, illustrated by white arrows for *Kn* = 0.387. With the higher rarefied degree of the gas, the secondary vortexes completely replace the main vortexes. The secondary vortexes individually exist near the corners of the wide channel, shown by white arrows for *Kn* = 3.87.

In terms of the temperature field, as the rarefied degree of the gas increases, the total number of gas molecules decreases. The thermal conductivity is weakened. Thus, the energy transmitting from the walls to the field decreases dramatically, as does the temperature difference. For different ratios of the gas mixtures with the same Knudsen number, the general distributions of the temperature field and the velocity field do not change dramatically with the variations of the ratio.

### 3.2. Temperature Gradient

In the DSMC method, the overall temperature of the gas T is as follows [38,39,40],

(5) $$T=\sum_{p=1}^{q}\left(\zeta_{p}T_{p}N^{\prime \prime }\right)/\sum_{p}^{q}\left(\zeta_{p}N^{\prime \prime }\right)$$

where *p* is the species of the simulation particle, *q* is the amount of the species of the simulation particle, $\sum N^{\prime \prime }$ is the weighted number of simulated molecules, and the temperature of species *p* is

(6) $$T_{p}=\left(3T_{\mathrm{tr},p}+\zeta_{\mathrm{rot},p}T_{\mathrm{rot},p}+\zeta_{\mathrm{vib},p}T_{\mathrm{vib},p}+\zeta_{\mathrm{el},p}T_{\mathrm{el},p}\right)/\zeta_{p}$$

where, $T_{\mathrm{tr},p}$, $T_{\mathrm{rot},p}$, $T_{\mathrm{vib},p}$, and $T_{\mathrm{el},p}$ are translational temperature, rotational temperature, vibrational temperature and electronic temperature, respectively. $\zeta_{\mathrm{rot},p}$, $\zeta_{\mathrm{vib},p}$, and $\zeta_{\mathrm{el},p}$ are the corresponding degrees of freedom. The effective number of degrees of freedom $\zeta_{p}$ of species *p* is

(7) $$\zeta_{p}=3+\zeta_{\mathrm{rot},p}+\zeta_{\mathrm{vib},p}+\zeta_{\mathrm{el},p}$$

Due to no consideration of the vibrational energy and the electronic energy ($\zeta_{\mathrm{vib},p}=\zeta_{\mathrm{el},p}=0$), the temperature of species *p* is

(8) $$T_{p}=\left(3T_{\mathrm{tr},p}+\zeta_{\mathrm{rot},p}T_{\mathrm{rot},p}\right)/\left(3+\zeta_{\mathrm{rot},p}\right)$$

where,

(9) $$T_{\mathrm{tr},p}=m_{p}\left\{\Sigma {\left(u_{p}^{2}\right)}^{\prime \prime }+\Sigma {\left(\nu_{p}^{2}\right)}^{\prime \prime }+\Sigma {\left(w_{p}^{2}\right)}^{\prime \prime }-u_{0}^{2}-\nu_{0}^{2}-w_{0}^{2}\right\}/\left(3k\right)$$

(10) $$T_{\mathrm{rot},p}=\left(2/k\right)\left(\Sigma \varepsilon_{\mathrm{rot},p}^{\prime \prime }/\zeta_{p}\right)$$

where, $\Sigma \varepsilon_{\mathrm{rot},p}^{\prime \prime }$ is the weighted sum of the rotational energy of the simulated molecules of species *p*.

By substituting Equations (9) and (10) into Equation (5), the overall temperature is obtained by the summation of all species of the simulation particles. Similarly, in the dsmcFoam, the overall temperature of the gas T is obtained by the mean value of the relevant macroscopic physical properties in the cell. The representation is as follows by dsmcFoam code [46],

(11) $$T=\frac{2\left(\rho_{\mathrm{lKE}}-0.5\rho_{m}U^{2}+\rho_{\mathrm{ilE}}\right)}{k\left(3\rho_{N}+\rho_{\mathrm{iDF}}\right)}$$

where $\rho_{\mathrm{lKE}}$ is mean value of the linear kinetic energy density, $\rho_{m}$ is the mean value of the mass density, U is the mean value of gas velocity,$\rho_{\mathrm{ilE}}$ is the mean value of the internal energy density, and $\rho_{N}$ is the mean value of the real gas-molecular number density in the cell. $\rho_{\mathrm{iDF}}$ is the mean value of the internal degree of the freedom density, but the influence of internal degree of freedom is not considered in the VHS model; here, it is assumed that $\rho_{\mathrm{iDF}}$ = 0. To simulate the molecular motion, the random numbers are generated in the DSMC method. The average values of macroscopic physical quantities are applied to maximize the simulation accuracy.

**Figure 6:**
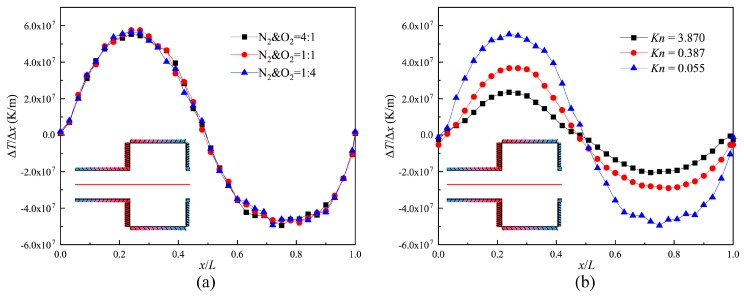
Temperature gradient on the central axis of the channel. (**a**) *Kn* = 0.055; (**b**) N_2_ and O_2_ = 4:1.

Figure 6 illustrates the distribution of the temperature gradient for three gas mixtures and three Knudsen numbers on the central axis of the channel (indicated by the red full line in Figure 6) within one structure unit. It can be seen that the changes in the composition of the gas mixtures do not make a difference in the distribution of the temperature gradient on the central axis. The distribution patterns are similar to the asymmetric sinusoid shown in Figure 6a. This is because the size of the narrow channel is much smaller, and more energy is transferred to the central axis.

Moreover, it was found that for different Knudsen numbers, these distribution patterns always remain as asymmetric sinusoidal patterns. The maximum temperature gradients respectively reach the medium points of the narrow channel and the wide channel (shown as Figure 6b). For the reason that the rarefied degree of the gas increases and thermal conductivity weakens, the maximal values of temperature gradient decrease with increasing Knudsen numbers. However, the velocity for *Kn* = 0.387 is larger than the velocity for *Kn* = 0.155 (Discussion later). This is because the thermal creep effect hardly appears near the slip regime. It is not obvious, though the temperature gradient is larger. Therefore, we conclude that the value of the temperature gradient does not correspond to the performance of the thermal creep effect near the slip regime.

### 3.3. Mean Velocity

**Figure 7:**
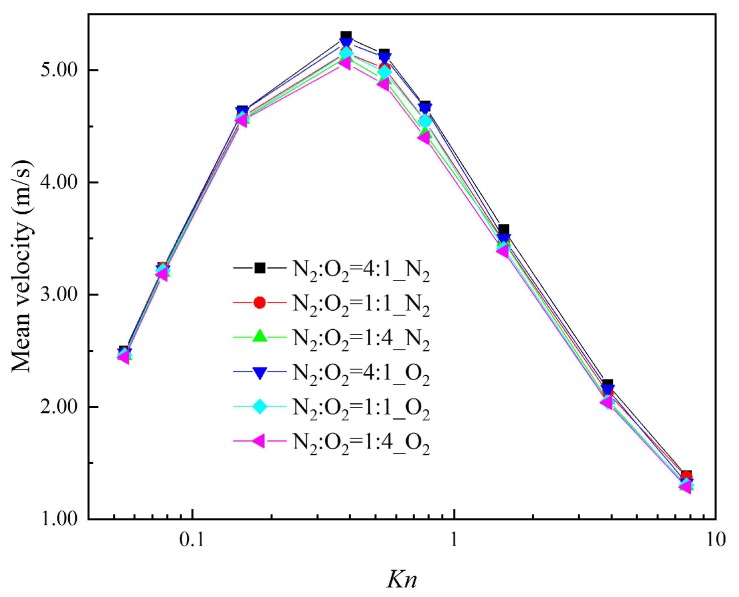
Mean velocities for different Knudsen numbers.

Figure 7 presents the mean velocity of each composition with the different gas mixture in a cross-section at *x* = 1 μm (indicated by the red full line in Figure 2) for different Knudsen numbers. Even in a mixture of N_2_ and O_2_ with a similar molecular mass, the velocity of N_2_ is larger than that of O_2_. The close relation between the thermal creep effect and the gas-molecular mass is clearly proved [60]. When the ratio of N_2_ rises in the gas mixtures, the thermal creep effect is enhanced. This not only causes *n* increase in the velocity of N_2_ itself, but also contributes to an increase in the velocity of O_2_. That is, N_2_ can promote the movement of O_2_.

It was also found that the mean velocity of each composition in different gas mixtures reaches the maximum for *Kn* = 0.387. When the gas mixtures are in the slip regime (*Kn* < 0.077) and free-molecular regime (*Kn* > 7.75), no matter how changeable the compositions of the gas mixtures are, the mean velocities of each composition are almost the same. That is because the thermal creep effect cannot be effectively induced by the temperature gradient in the slip regime, while in the free-molecular regime the thermal creep effect is weaker. Therefore, regarding systems that process gas separation using the thermal creep effect, it should be guaranteed that gas mixtures are in a transition regime in order to improve the efficiency and quality of gas separation.

### 3.4. Mass Flow Rate

During one physical time $t_{\mathrm{avg}}$ which used for calculating the average of the macroscopic physical quantities, the mass flow rate is obtained by calculating the total mass of all particles that pass through the cross section in *x* = 1 μm (indicated by a red full line in Figure 2). This is demonstrated by the following equations:

(12) $$M_{f}=\frac{\sum_{i}^{\Delta N}E_{p}m_{i}\mathrm{sgn}({\vec{c}}_{i}\cdot {\vec{n}}_{f})}{t_{\mathrm{avg}}}$$

(13) $$\mathrm{sgn}({\vec{c}}_{i}\cdot {\vec{n}}_{f})=\left\{ \begin{array}{ll} 1 & if\;{\vec{c}}_{i}\cdot {\vec{n}}_{f}>0\\ 0 & if\;{\vec{c}}_{i}\cdot {\vec{n}}_{f}=0\\ -1 & if\;{\vec{c}}_{i}\cdot {\vec{n}}_{f}<0 \end{array}\right.$$

where ΔN is the total number of the simulating particles passing through the cross section within $t_{\mathrm{avg}}$, $m_{i}$ is the mass of the molecules; ${\vec{c}}_{i}$ is the velocity of particles; ${\vec{n}}_{f}$ is the unit normal vector of the cross-section, and the positive and negative directions are the same as the *x*-axis. Likewise, in order to improve the accuracy of the simulation in the DSMC, the paper decreases the numerical error through the mean value of the mass flow rate for a large number of cycle numbers. The sampling interval is usually the same as the physical time, $t_{\mathrm{avg}}$.

**Figure 8:**
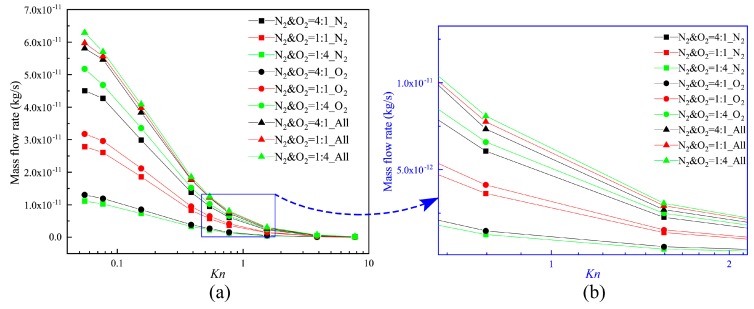
Mass flow rates for different Knudsen numbers.

Therefore, the total mass flow rates of these three different N_2_–O_2_ gas mixes can be obtained. The mass flow rate of each composition for the different Knudsen numbers is illustrated in Figure 8. Mass flow rate decreases with the increase of the Knudsen number, and the maximum and minimum values occur respectively at *Kn* = 0.055 and *Kn* = 7.75. This is mainly because the enhancement of the gas rarefied degree leads to a decrease in the number of molecules. The variation of the mass flow rate is the largest in the Knudsen number range from 0.155 to 0.387. It is determined by the stronger thermal creep effect within this range.

Furthermore, though O_2_ molecules are heavier and have higher densities in each of the gas mixtures, N_2_ has the larger flowing velocity, and the number of N_2_ gas molecules passing through the channel per unit time increases. Thus, the increase of the ratio of N_2_ weakens the influence of the differences in the value of the densities. This increases the mass flow rate to some degree. The thermal creep effect is stronger, especially when the value of the Knudsen number is in the range of 0.155–0.387. The total mass flow rates of these three N_2_–O_2_ gas mixtures are much closer to each other.

## 4. Conclusions

The flow characteristics of N_2_–O_2_ gas mixtures in the rectangular Knudsen pump are studied by using the DSMC method. By exerting temperature gradient boundary conditions on the walls, a thermally induced flow (thermal creep flow) is successfully generated. The influences of N_2_–O_2_ gas mixtures in different ratios, and different gas rarefied degrees (different Knudsen numbers) on the flow characteristics of gases are well studied. The following conclusions can be drawn:

(a) Under the same Knudsen number, the flow fields of the three different gas mixtures in the Knudsen pump channel are highly similar. The distribution of the temperature gradient is all asymmetric sinusoid in nature on the central axis of the channel. That is, the variations of the gas compositions do not make a difference in the distribution of the flow field in the Knudsen pump channels.

(b) Even in N_2_ and O_2_ gas mixtures with similar molecular masses, N_2_ is found to have a stronger thermal creep effect. It was successfully verified that the thermal creep effect has a relationship with the weight of the gas molecules.

(c) In gas mixtures, N_2_ has a larger velocity than O_2_. If the proportion of N_2_ increases, the overall velocity also increases. The lighter gas can promote the movement of the heavier gas.

(d) The lighter gas and heavier gas respectively correspond to a larger volume flow rate and a larger mass flow rate. With the ratio of the lighter gas increasing, the incremental volume flow rate somewhat weakens the difference of the mass flow rate resulting from the difference of densities. Even though the ratios of each composition of the gas mixtures differ greatly, the total mass flow rates are almost equal, especially when the thermal creep effect is the strongest.

## Figures and Tables

**Table 1:** The mass flow rate for different cell sizes and the numbers of the simulator in the cell, at the reference for *H* = 1 μm, *D* = 3 μm, *L* = 4 μm, *L_m_* = 2 μm, *T*_c_ = 225 K, *T*_h_ = 375 K and *Kn* = 0.155.

Parameter	Case 1	Case 2	Case 3	Case 4	Case 5	Case 6
Number of the cell *N_C_*	9248	5100	3200	3200	3200	3200
$\Delta x/\lambda_{\min}$	10/63	5/24	1/3	1/3	1/3	1/3
The number of the simulator in the cell *n*	15	15	10	15	20	30
Time step Δt(s)	4 × 10^−11^	5 × 10^−11^	1 × 10^−10^	1 × 10^−10^	1 × 10^−10^	1 × 10^−10^
Mass flow rate (10^−11^) (kg/s)	3.8294	3.8328	3.8681	3.8367	3.8359	3.8384

**Table 2:** Physical properties of N_2_ and O_2_ for *T*_0_ = 273 K.

Gas	Property			
	Molecular Mass *m* (10^−^^27^) (kg)	Molecular Diameter *d* (10^−^^10^) (m)	Internal Degree of Freedom	ω
N_2_	46.50	4.17	2	0.74
O_2_	53.12	4.07	2	0.77
